# Classification of Rectus Diastasis—A Proposal by the German Hernia Society (DHG) and the International Endohernia Society (IEHS)

**DOI:** 10.3389/fsurg.2019.00001

**Published:** 2019-01-28

**Authors:** Wolfgang Reinpold, Ferdinand Köckerling, Reinhard Bittner, Joachim Conze, René Fortelny, Andreas Koch, Jan Kukleta, Andreas Kuthe, Ralph Lorenz, Bernd Stechemesser

**Affiliations:** ^1^Wilhelmsburger Krankenhaus Groß-Sand, Hamburg, Germany; ^2^Department of Surgery, Center for Minimally Invasive Surgery, Academic Teaching Hospital of Charité Medical School, Vivantes Hospital, Berlin, Germany; ^3^Marienhospital Stuttgart, Stuttgart, Germany; ^4^Hernienzentrum, Munich, Germany; ^5^Department of General Surgery, Medical Faculty, Wilhelminen Hospital, Sigmund Freud University, Vienna, Austria; ^6^Hernia Center Cottbus, Cottbus, Germany; ^7^Visceral Surgery Zurich, Hirslanden Klinik Im Park, Zurich, Switzerland; ^8^DRK-Krankenhaus Clementinenhaus, Hanover, Germany; ^9^Hernia Center, 3+Chirurgen, Berlin, Germany; ^10^Hernienzentrum Köln, PAN–Klinik, Köln, Germany

**Keywords:** rectus diastasis, classification, concomitant hernia, pregnancy, defect width

## Abstract

**Introduction:** Recently, the promising results of new procedures for the treatment of rectus diastasis with concomitant hernias using extraperitoneal mesh placement and anatomical restoration of the linea alba were published. To date, there is no recognized classification of rectus diastasis (RD) with concomitant hernias. This is urgently needed for comparative assessment of new surgical techniques. A working group of the German Hernia Society (DHG) and the International Endohernia Society (IEHS) set itself the task of devising such a classification.

**Materials and Methods:** A systematic search of the available literature was performed up to October 2018 using Medline, PubMed, Scopus, Embase, Springer Link, and the Cochrane Library. A meeting of the working group was held in May 2018 in Hamburg. For the present analysis 30 publications were identified as relevant.

**Results:** In addition to the usual patient- and technique-related influencing factors on the outcome of hernia surgery, a typical means of rectus diastasis classification and diagnosis should be devised. Here the length of the rectus diastasis should be classified in terms of the respective subxiphoidal, epigastric, umbilical, infraumbilical, and suprapubic sectors affected as well as by the width in centimeters, whereby W1 < 3 cm, W2 = 3− ≤ 5 cm, and W3 > 5 cm. Furthermore, gender, the concomitant hernias, previous abdominal surgery, number of pregnancies and multiple births, spontaneous birth or caesarian section, skin condition, diagnostic procedures and preoperative pain rate and localization of pain should be recorded.

**Conclusion:** Such a unique classification is needed for assessment of the treatment results in patients with RD.

## Introduction

The rectus muscles are normally fused at the midline with no more than 1 to 2 cm separating them (1). Rectus diastasis (RD) (diastasis recti, divarication of the rectus muscles) is an acquired condition in which the rectus muscles are separated by an abnormal distance along their length, but with no fascial defect (1). A separation of >2 cm is considered to be a rectus diastasis (1). It is most commonly found in middle-aged and older men with central obesity, or in women who have carried a large fetus or twins to term (1). RD is characterized by a protruding midline following an increase in intra-abdominal pressure (2). The condition is characterized by a gradual thinning and widening of the linea alba, combined with a general laxity of the ventral abdominal wall muscles (2). The musculofascial continuity of the midline and subsequent absence of a true hernia sac is what sets RD apart from a ventral hernia (2). But thinning and stretching of the linea alba is an important risk factor for actual development of midline hernias (umbilical, epigastric, trocar, incisional hernia) due to the deterioration of the connective tissue and the pulling of the abdominal muscles (3). In a series of even small umbilical and epigastric hernias (<2 cm) concomitant rectus diastasis was diagnosed in 45% of patients (3). If RD is associated with midline hernias, the surgical procedure most recommended could be corrective surgery of both pathologies at the same time (4).

In systematic reviews the published literature on surgical treatment of RD is assessed to be of low scientific and methodological quality (2, 5). There is currently no consensus as to the definition and classification of RD (4). Based on the current literature, no clear distinction can be made in the recurrence rate, postoperative complications, or patient reported outcomes (2). This applies, in particular, to the numerous innovative minimally invasive techniques recently reported in the literature for treatment of RD with concomitant ventral hernias (4, 6–19). The promising results of new procedures with extraperitoneal mesh placement and anatomical restoration of the linea alba, such as the endoscopic-assisted or endoscopic mini open sublay repair (MILOS, EMILOS), endoscopic-assisted linea alba reconstruction (ELAR), laparoscopic linea alba stapler repair, enhanced total extraperitoneal ventral hernia repair (eTEP), laparoscopic intracorporeal rectus aponeuroplasty (LIRA), preaponeurotic endoscopic repair (REPA) and totally endoscopic sublay (TES), have to be confirmed in future trials (4, 6–19). For enhanced comparability of the treatment results of hernia surgery a recognized classification system is indispensable (20). Moreover, all patient characteristics influencing the treatment results should be analyzed (21).

Therefore, on the basis of the existing literature, a working group of the German Hernia Society (DHG) and the International Endohernia Society (IEHS) set about defining the patient- and technique-related factors in order to propose a classification of RD.

## Materials and Methods

**Figure 1:**
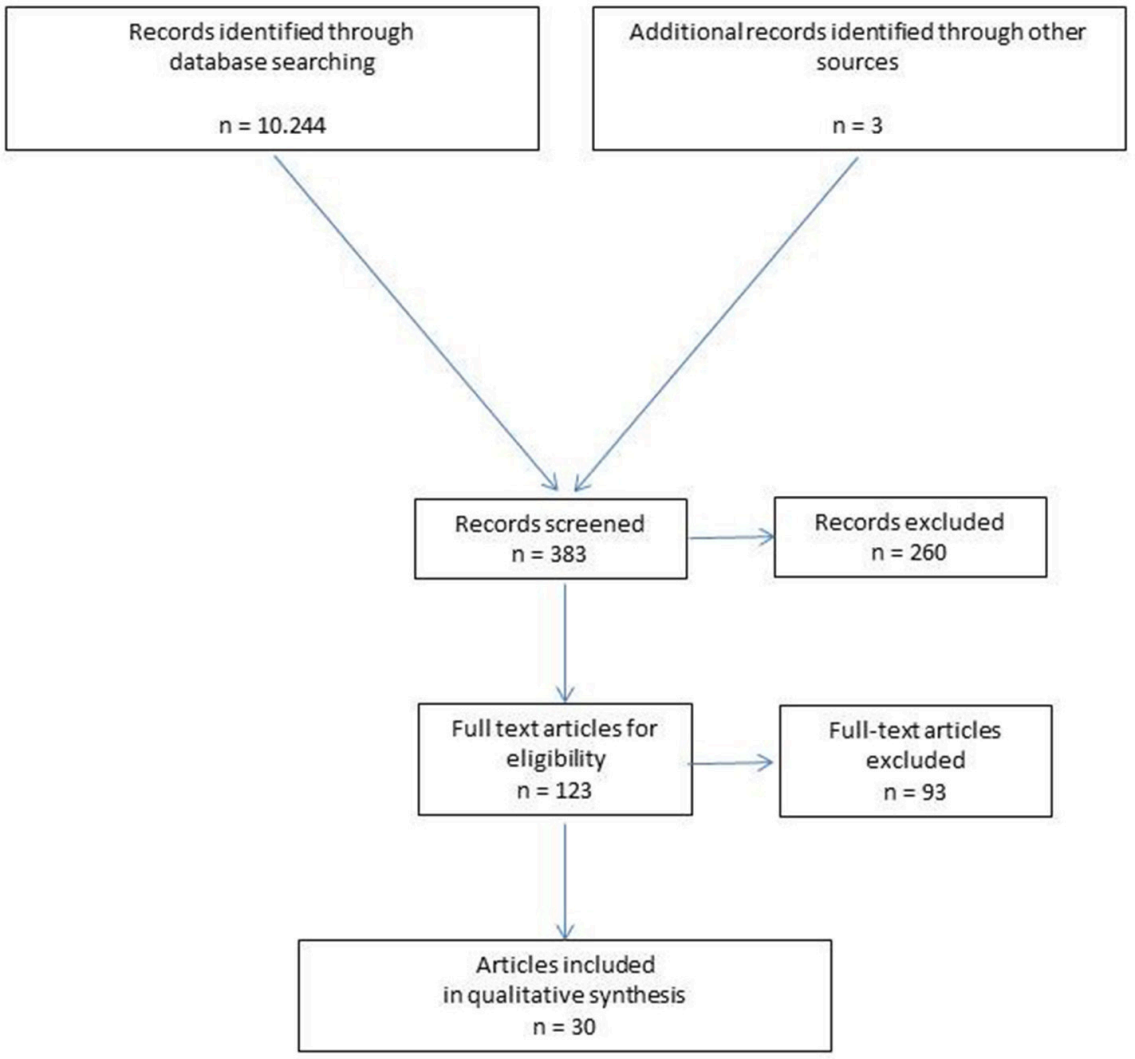
Flowchart of study inclusion.

The members of the working group were requested, on the basis of a literature search, to identify potential classification features for rectus diastasis. The results of that literature search were presented by the members to the group at a meeting in May 2018 in Hamburg. Then a consensus was reached by the group (Figure 1). The consensus-building results are now presented below. Consensus building was based on 30 relevant literature sources identified in searches of PubMed, Medline, Google scholar, and the Cochrane Library. The following search terms were used: “Rectus diastasis,” “Diastasis recti,” “Rectus divarication,” “Rectus abdominis diastasis,” “Sublay repair,” “Sublay technique,” “Retromuscular mesh placement,” “Ventral hernia,” “Ventral hernia repair,” “Abdominal wall reconstruction.”

## Results

### Normal Anatomy of the Linea Alba

The existence of the linea alba implies a physiological distance between the two rectus muscles. Beer et al. (22) examined 150 nulliparous women between 20 and 45 years of age and with a body mass index <30 kg/m^2^ by ultrasound at three reference points: the origin at the xiphoid, 3 cm above and 2 cm below the umbilicus. The mean width was 7 ± 5 mm at the xiphoid, 13 ± 7 mm above and 8 ± 6 mm below the umbilicus. For the definition of the normal width of the linea alba, the 10 and 90th percentiles were taken. The authors concluded that in nulliparous women the linea alba can be considered “normal” up to a width of 15 mm at the xiphoid, up to 22 mm at 3 cm above the umbilicus and up to 16 mm at the reference point 2 cm below the umbilicus.

In a recent ultrasound trial of 84 primiparous women, Mota et al. (23) measured the linea alba during and after pregnancy. The normal width of the linea alba was defined by the 20 and 80th percentiles. During pregnancy, the 20th and the 80th percentile corresponded to 49–79 mm at 2 cm below the umbilicus, 54–86 mm at 2 cm above the umbilicus and 44–79 mm at 5 cm above the umbilicus. At 6 months postpartum, the 20th and the 80th percentile corresponded to 9–21 mm, 17–28 mm, and 12–24 mm at 2 cm below, 2 cm above and 5 cm above the umbilicus, respectively.

In an anatomical study by Rath et al. (24), RD in patients below 45 years of age was considered as a separation of the two rectus muscles exceeding 10 mm above the umbilicus, 27 mm at the umbilical ring and 9 mm below the umbilicus. Above 45 years of age, the corresponding values were 15, 27, and 14 mm, respectively.

Hence, the anatomical measurements and ultrasound examinations produce similar findings. A separation of the rectus muscles of up to 2 cm can thus be considered normal. A separation of both medial edges of the rectus muscles of >2 cm must therefore be considered pathologic.

### Width of RD

Ranney (25) proposed a classification of RD based on the width of the defect (Table 1): An observed separation of <3 cm between the rectus muscles has been labeled mild diastasis, 3–5 cm separation moderate diastasis and more than 5 cm severe diastasis.

**Table 1:** Width of RD according to Ranney (25).

W1	<3 cm
W2	3 – ≤ 5 cm
W3	>5 cm

### Length of RD

**Figure 2:**
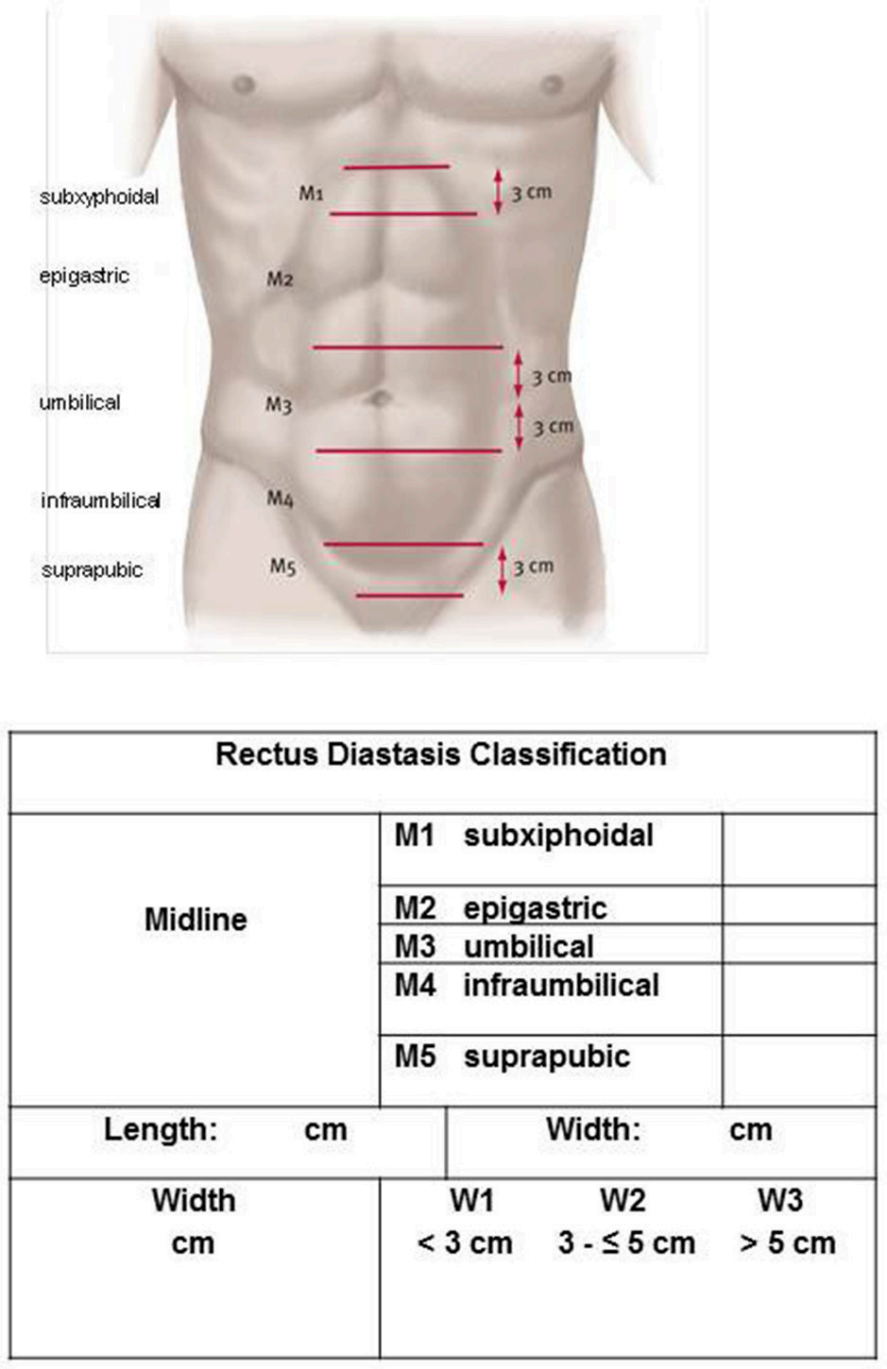
Length of RD according to the EHS incisional hernia classification.

For the length of the RD the European Hernia Society classification of midline incisional hernias can be used (20), separating the distance between the xiphoid and pubic bone into the subxiphoidal, epigastric, umbilical, infraumbilical, and suprapubic sectors (Figure 2). In addition to classification of the width and length (Figure 2 and Table 1), the maximum width and length of the RD should also be given in centimeters.

### Concomitant Hernias

Since concomitant hernias are the real indication for surgical treatment of RD (4), these should also be recorded. Often, several hernias are observed in association with RD (Table 2).

**Table 2:** Type of concomitant hernias in patients with RD.

Umbilical hernia	yes	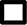	no	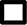
Epigastric hernia	yes	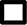	no	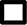
Port site hernia	yes	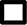	no	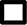
Incisional hernia	yes	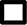	no	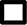

### Previous Operations

It is also important to document all previous laparoscopic or open surgical procedures in cases of rectus diastasis (Table 3). Here a distinction should be made between previous primary ventral hernia repairs (umbilical or epigastric hernia) and laparoscopic and open abdominal surgical procedures as well as gynecology operations (Table 3) (26).

**Table 3:** Previous abdominal surgery within the width and length of the RD.

No previous surgery	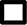
Previous laparoscopic primary ventral hernia repair	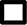
Previous open primary ventral hernia repair	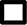
Previous other laparoscopic procedures	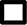
Previous other open procedure with midline incision	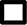
Previous other open procedure with lateral incision	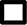
Previous cesarion section	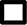

### Number of Pregnancies and RD

It is believed that women with RD have a greater number of pregnancies and deliveries. However, the results of other studies showed that RD above the umbilicus has similar prevalence in primiparae and multiparae (27). In order to obtain better data on this in the future, the number of pregnancies and multiple births should be precisely documented (Table 4). Older age at the first pregnancy and caesarian section lead more often to post-partum functional deficits of the lower trunk.

**Table 4:** Number of pregnancies and multiple birth.

1. Pregnancy		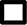		
2. Pregnancy		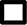		
3. Pregnancy		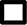		
4. Pregnancy		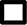		
> 4 Pregnancies		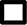		
Multiple birth		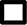	with 2 babies	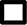
			with 3 babies	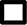
			with > 3 babies	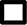

### Skin Condition

Following childbirth women often experience not just RD but are also left with considerable excess skin, with upright skin folds. These patients will not benefit from corrective RD surgery alone but will also need to undergo abdominoplasty (28). Without appropriate abdominoplasty there will be even more excess skin and more pronounced skin folds following corrective RD surgery alone, with an unaesthetic cosmetic result and corresponding patient dissatisfaction. Therefore, due attention should be paid to the skin condition in therapy decision-making (Table 5) (28).

**Table 5:** Skin condition.

S0	No skin laxity and no skin folds	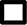
S1	Minor skin laxity and only few skin folds	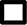
S2	Major skin laxity and extreme skin folds	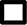

### Diagnosis of RD

In a systematic review the available information supports the belief that ultrasound and calipers are adequate methods to asses RD (29). For other methods limited measurement information of low to moderate quality is available and further evaluation of these measurement properties is required (Table 6) (29).

**Table 6:** Diagnostics of RD.

Clinical bulge while standing	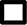
Clinical bulge at sit-up	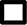
Calipers	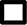
Ultrasound	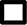
MRI	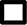
CT	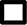
Intraoperative measurement	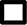

### Preoperative Pain

Patients with ventral hernias who already experience preoperative pain are also at higher risk of postoperative chronic pain (21). Patients with RD also experience more low back pain due to the instability of the abdominal wall (30). RD width may be associated with severity of low back pain (30). Therefore, the preoperative pain severity and localization should be carefully documented (Tables 7, 8).

**Table 7:**
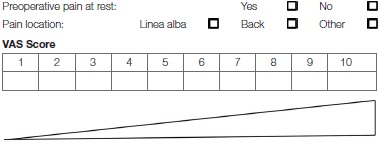
Preoperative pain at rest.

**Table 8:**
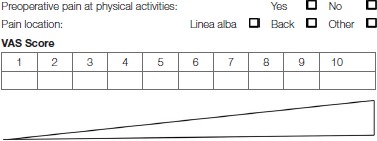
Preoperative pain at physical activities.

### Other Influencing Factors on the Outcome of Hernia Surgery

In addition to the aforementioned, specific characteristics of rectus diastasis that could potentially affect the outcome, all other patient- and technique-related factors known to impact the outcome should of course also be documented (Tables 9, 10) (21).

**Table 9:** Patient-related influencing factors on the outcome in hernia surgery.

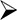 Gender
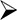 BMI
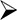 ASA score
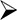 Age
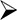 Risk factors
(COPD, Diabetes, Aortic aneurysm, Immunosuppression, Corticoids, Smoking, Coagulopathy, Antiplatelet medication, Anticoagulation therapy)

**Table 10:** Technic-related influencing factors on the outcome in hernia surgery.

Mesh type
Mesh size
Defect closure
Mesh-fixation – Suture, Tacker, Glue, Combination

## Discussion

In systematic reviews of the surgical treatment of RD, the published studies are assessed to be of low scientific and methodological quality (2, 5). The main reason for the poor quality of these studies on surgical treatment of RD is thought to be the lack of a uniform definition and classification of RD. This hampers comparability of the study findings on RD. Recently, numerous innovative techniques, with case series reports, have been introduced for surgical treatment of RD (4, 6–19). Classification of RD is needed in order to be able to evaluate the significance of all these different techniques and to properly characterize the respective patient collective in comparative studies. Only on the basis of comparative RD patient collectives can an acceptable method comparison be made.

Therefore, a working group of the DHG and the IEHS has compiled the classification presented here. In doing so, it has closely followed the European Hernia Society classification of primary and incisional abdominal wall hernias (20) and has added specific aspects of RD. In addition to the assignment of the RD longitudinal extension to midline sectors (subxiphoidal, epigastric, umbilical, infraumbilical, and subrapubic), the extension in centimeters is also recorded, as is the width of the rectus diastasis. Besides, the width of the rectus diastasis should be classified according to Ranney (25). An observed separation of <3 cm between the rectus muscles is labeled mild diastasis, 3–5 cm separation of the rectus muscles moderate diastasis and more than 5 cm severe diastasis (25). The concomitant hernias should be documented as well as any previous surgery. In particular in the case of women, the skin condition should be described since this has implications for treatment, generally involving a combination of corrective rectus diastasis surgery and abdominoplasty (28). The number of pregnancies and multiple births should also be documented. Since preoperative pain constitutes a risk factor for chronic postoperative pain, preoperative pain severity, and pain localization should also be noted (21). The diagnostic procedures used should also be specified in order to ascertain the basis on which the aforementioned parameters were defined.

In addition to these RD-specific factors, all patient-related factors known to impact the outcome of hernia surgery should of course also be documented (age, gender, ASA score, BMI, risk factors, COPD, diabetes, aortic aneurysm, immunosuppression, corticoids, smoking, coagulopathy, antiplatelet medication, and anticoagulation therapy) (21).

Likewise, the technical details of the operation, such as the mesh type, mesh size, defect closure and the fixation technique, should be documented (21). Only in that way will it be possible in multivariable analyses to identify all influencing factors on the outcome of a specific operation technique. Precise characterization of the patient collective is also needed for comparative analysis of operation techniques (21). Armed with that information it will be possible in the future to make a quality-oriented decision on the many new surgical techniques for treatment of RD.

In summary, in addition to the patient- and technique-related factors, RD-specific classification is needed for qualitative assessment of the numerous innovative surgical techniques for treatment of RD. The proposal presented here by a working group of the DHG and the IEHS classifies RD on the basis of the diastasis length (subxiphoidal, epigastric, umbilical, infraumbilical, and suprapubic), diastasis width (W1 < 3 cm, W2 = 3 – ≤ 5 cm, and W3 > 5 cm) concomitant hernias (umbilical, epigastric, port-site, and incisional hernia), previous operations, number of pregnancies and multiple births, skin condition and severity and localization of preoperative pain.

## Author Contributions

WR and FK: publication concept and draft. All authors: literature search, literature analysis, and critical review of the publication draft.

### Conflict of Interest Statement

The authors declare that the research was conducted in the absence of any commercial or financial relationships that could be construed as a potential conflict of interest.
